# Anodic behaviour of Cu, Zr and Cu–Zr alloy in molten LiCl–KCl eutectic

**DOI:** 10.1098/rsos.181278

**Published:** 2019-01-09

**Authors:** Yanqing Cai, Xinggang Chen, Qian Xu, Ying Xu

**Affiliations:** 1 Hebei Provincial Key Laboratory of Inorganic Nonmetallic Materials, College of Material Science and Engineering, North China University of Science and Technology, Tangshan, People's Republic of China; 2 School of Materials Science and Engineering, Shanghai University, Shanghai 200072, People's Republic of China

**Keywords:** anodic dissolution, potential, Cu–Zr alloy, polarization curve, molten salt

## Abstract

The anodic dissolution behaviours of Cu, Zr and Cu–Zr alloy were analysed in LiCl–KCl at 500°C by anode polarization curve and potentiostatic polarization curve. The results show that the initial and fast-dissolving potentials of Cu are −0.50 and −0.29 V, and Zr are −1.0 and −0.88 V, respectively. But, in the Cu–Zr alloy, the initial and fast-dissolving potentials of Cu are −0.52 and −0.41 V, and Zr are −0.96 and −0.92 V, respectively. The potentials satisfy the selection dissolution principle that Zr in the alloy dissolves first, while Cu is left in the anode and is not oxidized. The passivation phenomenon of Zr is observed in the quick dissolution of Zr, while it is not observed in the Cu–Zr alloy. Moreover, from the above anodic dissolution results, potentiostatic electrolysis of Cu–Zr alloy was carried out at −0.8 V for 40 min, and the anodic dissolution mechanism and kinetics of Zr in Cu–Zr alloy were also discussed. In the initial stage, Zr dissolves as Zr^4+^ ions from the alloy surface and enters into the molten salt, leaving a Cu layer called ‘dissolving layer’ on the surface of the alloy. After that, another layer between the matrix and ‘dissolving layer’ called ‘diffusion–dissolution layer’ appears. Zr diffuses in the alloy matrix and dissolves as Zr^4+^ ions on the surface of the ‘diffusion–dissolution layer’ continuously, and Zr^4+^ ions diffuse through the ‘dissolving layer’ and enter into the molten salt finally. In addition, the factors affecting the dissolution of Cu–Zr alloy, such as time and potential, were also investigated. The dissolution loss increases with the increasing dissolution potential and time, while the dissolution rate increases with the increasing dissolution potential and declines with the prolonging dissolution time.

## 1. Introduction

Nuclear energy, as a kind of clean energy, has the characteristics of high efficiency, safety and economy. Moreover, with the expected depletion in the supplies of fossil fuels throughout the world, a shift toward nuclear energy seems inevitable [1]. Nuclear-grade zirconium, used in nuclear reactors for structural purposes and for containing the nuclear fuel, is an irreplaceable component because of its very low neutron capture cross-section. The nuclear power industry consumes around 90% of the zirconium prepared each year, and the demand for nuclear-grade is expected to increase dramatically [2].

Zr alloys, which are widely used as cladding hull materials for light water reactors, contain approximately 98% of Zr with other alloying elements such as Sn, Fe and Cr for zircaloy-4 and Sn, Nb and Fe for Zirlo [3]. However, after being used for a certain number of years, Zr alloys would achieve its service life due to wear, corrosion and other causes, resulting in a large amount of scrap Zr alloy [4]. Depending on its radioactivity and the pollution to the environment, scrap Zr alloy would be disposed of as intermediate or low-level waste, causing serious problems for storage and handling. Moreover, the nuclear-grade Zr in the scrap alloy, which is nearly free of hafnium, is expensive and very difficult to acquire, so it is a pity to throw it away without repurposing. Thus, the recovery of Zr is an alternative option for a substantial reduction of the hull waste, which can be re-used as an additional fuel component for nuclear reactors and so on, and can also avoid the environmental pollution problems.

The electrochemical refining method in the molten chlorides has been developed as a promising option for the zirconium refining from impure zirconium metal, alloy or spent metal fuels [2,3]. A few papers have been published on the electrorefining of zirconium from zircaloy-4 cladding hulls in LiCl–KCl–ZrCl_4_ molten salts [4–9], and the purity of the recovered Zr deposits from zircaloy-4 appears to be 99.94 wt%. However, there were also some problems in the electrorefining process of Zr alloy. Firstly, the anode used in the electrorefining was solid, and the diffusion of Zr is difficult and complex. Secondly, the starting zirconium compound, ZrCl_4_, usually sublimates at 331°C, and some will be lost if it is directly introduced into the salt. Therefore, a low-melting point Cu–Sn–Zr alloy or Cu–Zr alloy serving as a liquid anode is proposed by our research group, while the highly pure zirconium can be deposited on the cathode, and the electrorefining process preformed in *in situ* prepared LiCl–KCl–ZrCl_4_ molten salt, which has been reported in our further works [10–12]. Actually, Zr in the Cu–Sn–Zr alloy and Cu–Zr alloy is spent zircaloy, while it is pure Zr in the present study for simplifying the study. The method would not only reduce the operating temperature but also provide a low-cost and semi-continuous process for the production of the nuclear-grade zirconium.

The electrorefining of Zr alloy should be carried out with Cu–Sn–Zr alloy or Cu–Zr alloy as an anode, while Sn has a low solubility in the alloy (6.2 wt%), so the investigation on the anode dissolution process of Cu–Zr alloy is necessary. Hence in this work, the electrochemical behaviours of Cu, Zr and Cu–Zr alloys were taken up to study the anodic dissolution process. A few papers [13–19] have been published earlier on the anodic dissolution of spent nuclear fuels, such as U–Pu–Zr, U–Zr fuels, and the dissolution potentials of U, Pu, Zr and U–Pu–Zr alloy were analysed. However, we have not come across any literature where anode polarization curve and potentiostatic polarization curve have been used to study the kinetics of dissolution of Cu–Zr alloy in the molten salt.

In this work, the anodic dissolution potentials of Cu, Zr and Cu–Zr alloy were analysed in LiCl–KCl at 500°C by cyclic voltammetry and potentiostatic polarization curve. Then, the potentiostatic electrolysis of Cu–Zr alloy was carried out at various potentials and different times. The anodic dissolution mechanism and kinetics of Zr in Cu–Zr alloy were also discussed.

## 2. Experimental set-up

### 2.1. Electrochemical chemicals and apparatus

All chemicals used in this work were of analytical grade. A mixture of 400 g LiCl–KCl eutectic salt (59 : 41 mol%) was used as the electrolyte, which was dried for more than 72 h at 300°C to remove residual water and then melted under argon atmosphere in an alumina crucible. To eliminate the residual water and other possible redox-active impurities, pre-electrolysis of the LiCl−KCl eutectic was conducted at 2.8 V for 2.0 h between two graphite rods prior to the electrochemical experiments. Pre-electrolysis and the following dissolution experiments were all conducted at 500°C. Copper wire (99.99% purity), silver wire (99.999% purity) and zirconium wire (sponge zirconium, 99.5% purity), supplied by Rare Metallic Co., Ltd of Shenyang, were used in the following experiments after successively polishing with sandpaper and washing with alcohol. Cu–Zr alloy was formed by melting copper powders and zirconium chippings according to the weight proportion of 47 : 53 at 1000°C, and the content of zirconium in the alloy was 53 wt%.

All of the electrochemical experiments were conducted under a high-purity argon atmosphere. A three-electrode electrochemical cell was assembled in an alumina crucible, which was positioned in a stainless steel vessel and heated with an electric furnace [10]. A graphite rod (spectrum pure) with a diameter of 14.0 mm served as the counter electrode (CE), which was provided by the Shanghai new graphite material Co., Ltd of Sinosteel Corporation (Shanghai, China). Copper wire, zirconium wire and Cu–Zr alloy rods, with diameters of 3.0, 3.0 and 4.0 mm, respectively, were used as the working electrodes (WE) for the investigation of anodic dissolution behaviours. The active electrode surface area was determined by measuring the immersion depth of the electrode in the salt after each experiment. The reference electrode (RE) was LiCl−KCl−1.0 wt% AgCl molten salt placed in a close-ended mullite tube with a 1.0 mm (dia.) silver wire immersed in it for electrical connections. All potentials in this work will be referred to this electrode unless otherwise stated.

An AUTOLAB/PGSTAT320 potentiostat from M/s. EcoChemie, The Netherlands, controlled with GPES 4.9 software for anode polarization curve and potentiostatic polarization curve, was used to conduct the electrochemical tests. An X-ray diffractometer (XRD) was used to characterize the metal and alloys. In addition, the interface morphology of alloy after the dissolving process was also measured by a scanning electron microscope (SEM, JMS 6480, JEOL company) and an optical microscope (OM, Axio Imager M2m type).

### 2.2. Anodic dissolution process

After pre-electrolysis, copper wire was immersed into the LiCl−KCl molten salt as the working electrode, and then anode polarization curve and potentiostatic polarization curve were measured by AUTOLAB/PGSTAT320 potentiostat. The anodic dissolution process was investigated to confirm the anodic dissolution potential of Cu. Similarly, the anodic dissolution process of Zr and Cu–Zr alloy was also investigated with zirconium wire, Cu–Zr alloy rods served as working electrodes. Special attention was paid to the dissolution potential of Cu–Zr alloy. After that, the anodic dissolution of Cu–Zr alloy was investigated by potentiostatic electrolysis at −0.8 V, and the interfacial morphology and constituent after dissolution were studied in detail to ascertain the anodic dissolution mechanism of Zr in Cu–Zr alloy. Moreover, the factors affecting the dissolution of Cu–Zr alloy, such as time and potential, were also studied.

## 3. Results and discussion

### 3.1. Determination for the anode dissolution potential of Cu

Steady-state anode polarization curve and potentiostatic polarization curve of Cu were performed for determining the anode dissolution potential of Cu.

**Figure 1.**
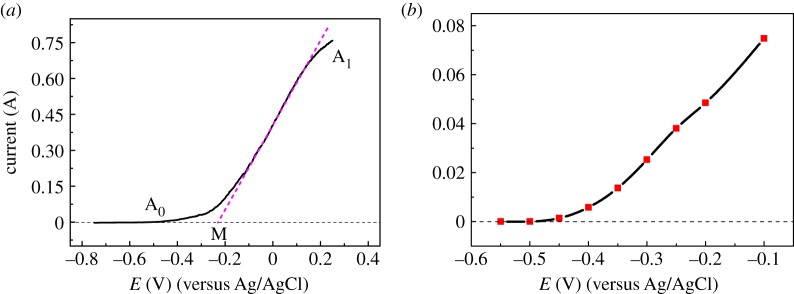
(*a*) Potentiodynamic anodic polarization curve and (*b*) potentiostatic anodic polarization curve of Cu in LiCl−KCl molten salt at 500°C, *S* = 0.628 cm^2^.

Firstly, the steady-state polarization curve of Cu in the anodic dissolution process was measured by potentiodynamic linear scanning at a low speed of 0.002 V s^−1^, as shown in figure 1*a*. The curve has the typical characteristics of metal anodic dissolution. When the anode polarization potential is higher than −0.51 V (A_0_), Cu begins to dissolve. The anode current increases as the scanning potential increases from −0.51 V to 0.15 V, which can be called the active dissolution area (A_0_A_1_) of Cu. In addition, an intersection M is obtained by the tangent of the anodic polarization curve and the horizontal line through the origin, which can deduce the fast-dissolving potential of Cu (−0.29 V). Once the polarization potential is more than this value, metal Cu begins to dissolve quickly. From the curves, we can know that Cu directly oxidizes to Cu^2+^ in the anode dissolution process [10].

Secondly, potentiostatic polarization curve was also measured to determine the anodic dissolution process of Cu. Stable values of the polarization current at potentials from −0.55 to −0.1 V were at −0.55, −0.5, −0.45, −0.4, −0.35, −0.3, −0.25, −0.2, −0.1 V, as shown in figure 1*b*. It shows that the initial anode dissolution potential of Cu is at about −0.50 V, which is consistent with the result obtained by the steady-state polarization curve. Although the measurement result is close to that of the steady-state value, the measurement needs a longer time. Thus, steady-state polarization curve measurement will be used for measuring the anode dissolution potentials of Zr and Cu–Zr alloy in the following study.

### 3.2. Determination for the anode dissolution potential of Zr

**Figure 2.**
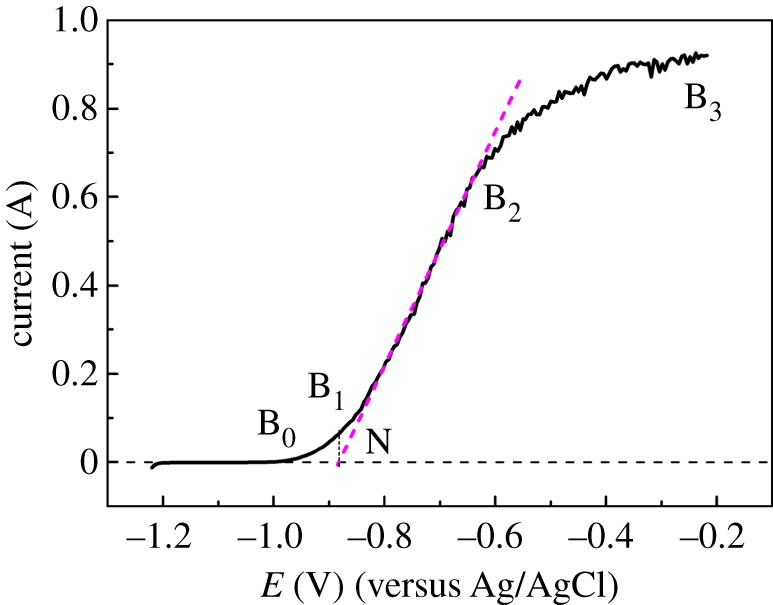
Potentiodynamic anodic polarization curve of Zr at 500°C, *S* = 0.628 cm^2^.

The steady-state polarization curve of Zr in the anodic dissolution process was measured to determine the anode dissolution potential by potentiodynamic linear scanning at a low speed of 0.002 V s^−1^, as shown in figure 2. The curves of Zr and Cu have the same characteristics. When the anode polarization potential is higher than −1.0 V (B_0_), Zr begins to dissolve. The anode current increases as the scanning potential increases from −1.0 V to −0.2 V, which can be called the active dissolution area (B_0_B_3_) of Zr. In addition, an intersection N is obtained by the tangent of anodic polarization curve and the horizontal line through the origin, which can deduce the fast-dissolving potential of Zr (−0.88 V), and the quickly dissolved region was B_1_B_2_. Once the polarization potential is more than −0.88 V, Zr begins to dissolve quickly. It is consistent with the anode dissolution principle of Zr, i.e. Zr would oxidize into Zr(IV) directly, this has been investigated in our former works [13,14].

**Figure 3.**
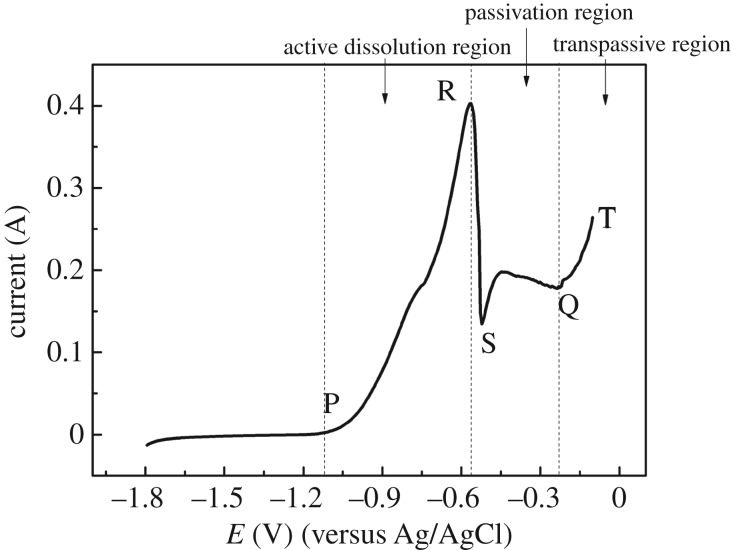
Linear sweep voltammetry curves in the anodic dissolution process of Zr (*S* = 0.628 cm^2^) at 500°C, scan range was from −1.80 V to −0.10 V, scan rate: 0.05 V s^−1^.

Linear sweep voltammetry was also developed from −1.80 to −0.1 V at a speed of 0.05 V s^−1^ to observe the anodic dissolution of Zr, as shown in figure 3. When the scanning potential increases to −1.10 V, the oxidation current begins to increase, indicating that Zr begins to dissolve. Then the oxidation current of zirconium increases gradually with the potential shifting positively, and an oxidation current peak appears at −0.65 V (R). The PR region can be called active dissolution region. After that, the current decreases rapidly to −0.60 V (S), this represents that the passivation phenomenon of Zr happens in the anodic dissolution process.

When the potential continually shifts to −0.25 V (Q), the oxidation current does not increase, so SQ is called the passivation region and the dissolution of Zr nearly stops. However, the current begins to increase with the potential scanning positively (QT), suggesting that the anodic dissolution of Zr goes into a transpassive region and anodic dissolution of Zr continues. To sum up, if Zr needs to dissolve quickly when it acts as an anode, we need to control the dissolution potential in the active dissolution region (PR), that is, in the range of −0.60 to −1.10 V.

The transformation of metal from active state to passive state is a very complicated process, and has not formed a complete theory so far. Currently, the phase membrane theory and adsorption film theory are more accepted by researchers [19]. The first theory insists that the solid products with good coverage are generated on the surface layer in the metal dissolving process, which can be considered as an independent phase (a film) and isolates the metal surface and solution. The second theory considers that an adsorption layer is formed on the metal surface, which is made up of oxide particles (in aqueous solution) or chlorides (in the molten chlorides). These particles change the structure of the metal–solution interface, making the reaction activation energy significantly increase. The commonality of two theories is that a very thin film was generated on the surface of the metal and hindered the dissolution of metal.

According to the metal passivation principle, the passivation that occurs in the anodic dissolution process should be caused by a film generated on the surface of the Zr metal. According to the literature [1,2], Zr can be oxidized to Zr(IV) in the anodic dissolution process, while the solubility of Zr(IV) in LiCl–KCl molten salt is limited (no more than 0.013 mol%). When the concentration of Zr(IV) is more than this value, Zr(IV) will combine with Cl^−^ and ZrCl_6_^2−^ complex will be generated. ZrCl_6_^2−^ is insoluble and adsorbs on the surface of Zr metal and forms a barrier when Zr dissolves continually and the concentration of Zr(IV) exceeds the solubility, leading to the passivation of Zr.

**Figure 4.**
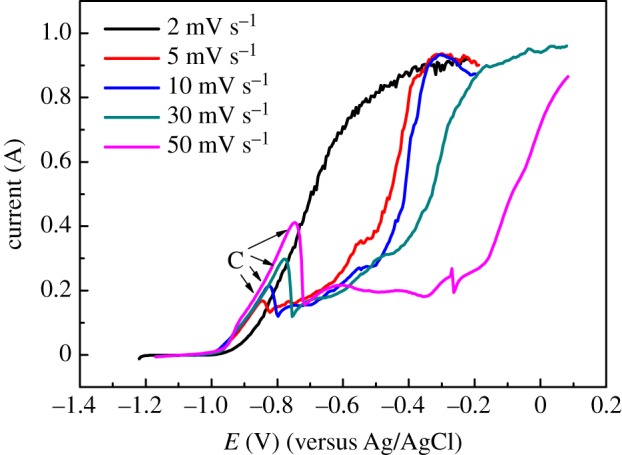
Potentiodynamic anodic polarization curve of Zr with various scan rates at 500°C, *S* = 0.628 cm^2^.

From figures 2 and 3, we can know that anodic dissolution phenomena of Zr have obvious differences when the scanning speeds are 0.002 and 0.05 V s^−1^. When the scanning speed is 0.002 V s^−1^, the passivation phenomenon does not appear, while at 0.05 V s^−1^, passivation phenomenon happens and anodic peak R appears at about −0.65 V. Therefore, anode polarization curves were measured at different scanning speeds to investigate the anodic dissolution process of Zr, as shown in figure 4. As the scanning speed increases, the current of peak C increases and the peak potential shifts positively, indicating that the passivation phenomenon becomes more obvious. What is more, the above results show that the faster the scanning speed, the faster the Zr dissolution into Zr(IV). Similarly, the generated ZrCl_6_^2−^ was easier to be saturated, and a film was generated on the surface of the Zr metal more quickly, causing a more obvious passivation phenomenon [1].

### 3.3. Determination for the anode dissolution potential of Cu–Zr alloy

**Figure 5.**
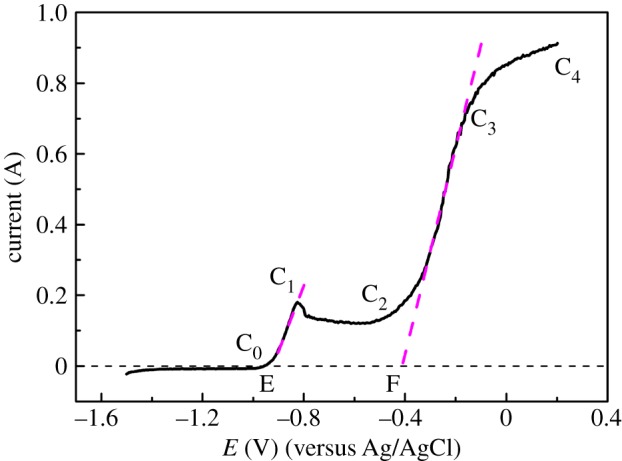
Potentiodynamic anodic polarization curve of Cu–Zr alloy at 500°C, *S* = 0.628 cm^2.^

Figure 5 shows the steady-state polarization curve of Cu–Zr alloy in the anodic dissolution process, which was obtained by potentiodynamic linear scanning at a low speed of 0.002 V s^−1^. As shown in figure 5, the current increases gradually and the alloy begins to dissolve at −0.96 V (C_0_), which corresponds to the dissolution of Zr in the Cu–Zr alloy. With the potential increasing to −0.82 V, the current reaches to the maximum (−0.18 A) and an anodic current peak C_1_ appears. Similarly, fast-dissolving potential of Zr in the alloy is obtained by the intersection E, and the value was −0.92 V. Then the current decreases slightly and becomes stable in a certain range.

There are two reasons for the appearance of extreme value: one is that the passivation of Zr occurs in the process of anodic dissolution reaction, and the passivation occurs at a lower scanning speed. The other is that Zr on the surface of Cu–Zr alloy has dissolved completely in the anodic dissolution process, and the diffusion of Zr from internal to the alloy surface is needed to ensure the dissolution of Zr continually.

With the potential shifting positively, the current begins to increase from −0.52 V (C_2_) after a period of stable stage, and the increasing rate slows down at about −0.16 V (C_3_). The fast-dissolving potential of Cu in the alloy is concluded by the intersection F, which was obtained by the tangent of anodic polarization curve and the horizontal line through the origin, and the value was −0.41 V. What is more, the reason for the slowing down dissolving rate from −0.16 V is that the characteristic of diffusion control in the polarization curve appears, and dissolution process is mainly controlled by the diffusion step.

### 3.4. Comparison of the anode dissolution potential of Cu, Zr and Cu–Zr alloy

**Figure 6.**
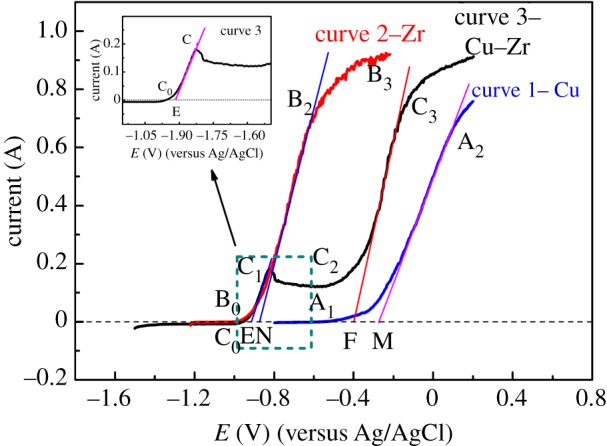
Comparison of the steady-state polarization curves of Cu, Zr and Cu–Zr alloy, scan rate: 0.002 V s^−1^.

Steady-state polarization curves of Cu, Zr and Cu–Zr alloy were combined and shown in figure 6 for comparing their anode dissolution potentials. By comparison, from curve 1 and curve 2, we can find that there is a large potential difference between Cu and Zr. So, the potentials satisfy the selection dissolution principle that Zr in the alloy should be dissolved firstly while Cu is left in the anode and does not oxidize. As seen from curve 3 in figure 6, when the potential was in the range from −0.90 to −0.80 V, the dissolution of Zr is very significant, and the passivation of Zr and the anodic dissolution of Cu did not occur. Therefore, potentiostatic electrolysis experiment could be carried out at −0.80 V to dissolve Zr, so that Cu is left in the alloy. The research about anodic dissolution potentials of Cu, Zr and Cu–Zr alloy provides a theoretical basis for the dissolution of Zr in the alloy and also leads to a theoretical foundation for electrolytic refining of Zr.

### 3.5. Anodic dissolution of Cu–Zr alloy by potentiostatic electrolysis

#### 3.5.1. Potentiostatic anodic dissolution of Cu–Zr alloy at −0.80 V

**Figure 7.**
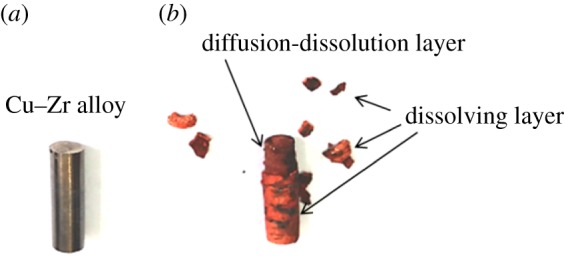
Photos of (*a*) Cu−Zr alloy and (*b*) alloy after electrolysis at −0.80 V for 40 min.

According to the anodic dissolution potentials obtained in figure 6, potentiostatic electrolysis of Cu–Zr alloy was carried out at −0.80 V. The photos of the Cu–Zr alloy before and after potentiostatic electrolysis at −0.80 V for 40 min are shown in figure 7. The colour of Cu–Zr alloy before electrolysis, as shown in figure 7*a*, is grey with metallic lustre similar to steel. After electrolysis, as shown in figure 7*b*, the grey colour with metallic lustre disappears, leading to a layer of loose red substances left on the surface, which can be called ‘dissolving layer’. The binding force between the red substance layer and the substrate is poor and some of them fall off. Moreover, except the ‘dissolving layer’, there was another layer between the dissolving layer and matrix called ‘diffusion–dissolution layer’.

Depending on the colour change of alloy, we can speculate that Cu and Zr are uniformly distributed on the surface and internal, while Zr dissolved from the surface and entered into the molten salt after electrolysis, and the Cu layer was left on the surface of the alloy. As Cu is red, the colour of alloy also might change from grey to red. Owing to the dissolution of Zr, the left Cu has a certain thickness and existed in a loose layer. After cooling, washing, weighing, etc., the loose dissolution layer on the alloy easily falls off due to the poor binding force.

**Figure 8.**
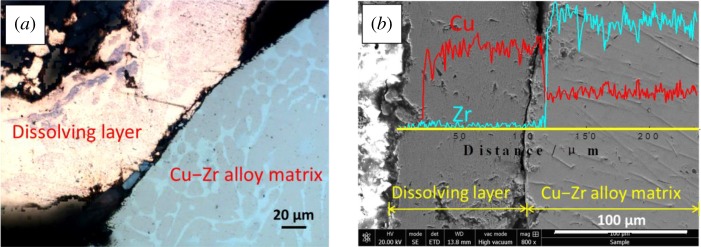
(*a*) Metallograph, (*b*) SEM images and the linear-EDS analysis of Cu–Zr alloy cross-section after electrolysis at −0.80 V for 40 min.

Metallographic microscope and SEM were used to observe the morphology of the alloy substrate (without dissolving) and the dropped dissolving layer after electrolysis at −0.8 V for 40 min, as shown in figure 8. We can see that the thickness of the layer is about 100 µm, and from the metallograph, we can see that there was a large difference in colour between the dissolving layer (red) and the matrix (white and pink interweaved). The linear-EDS analysis was carried out on the yellow line area in figure 8*b*; the result shows that the dissolving layer is almost all Cu, and no Zr is left; the proportion of Cu and Zr in the matrix remains fixed. The result indicates that Zr has dissolved from dissolving layer and entered into LiCl–KCl molten salt. In addition, some loose pore structures were found in the dissolving layer, which should be the dissolved region of Zr from the alloy.

**Figure 9.**
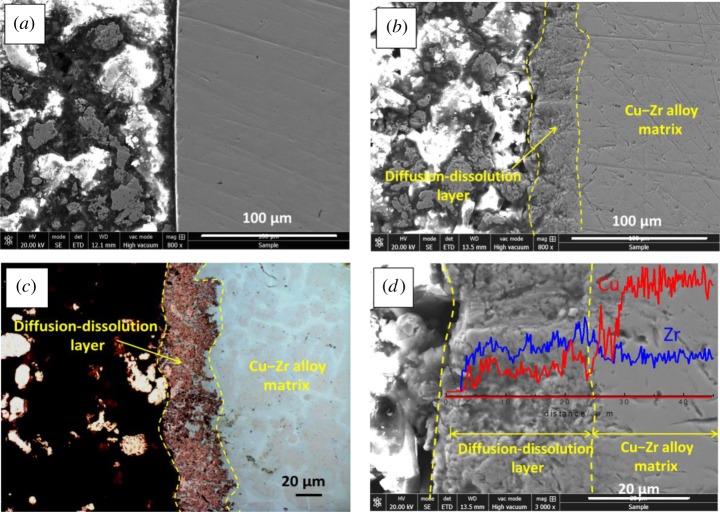
SEM images and the linear-EDS analysis (*a,b,d*) and metallograph (*c*) of Cu−Zr alloy cross-section before and after electrolysis at −0.80 V for 40 min.

Moreover, ‘diffusion–dissolution layer’ between the dissolving layer and matrix is shown in figure 9. Figure 9*a* shows that the edge of Cu–Zr alloy before dissolving is smooth. After electrolysis at −0.80 V for 40 min, the edge shown in figure 9*b–d* is the ‘diffusion–dissolution layer’, which has become rough with a certain thickness of 30–40 µm.

As shown in figure 9*c*, from the metallograph of Cu−Zr alloy cross-section after electrolysis, we can deduce that most of the red particles are the residual Cu. Also, there is also some light white area, which is similar to that in the matrix and should be the undissolved Zr or Cu–Zr alloy. In addition, the linear-EDS analysis was carried out to verify the deduction. From figure 9*d*, we can find that the content of Zr in the diffusion–dissolution layer is lower than that in the matrix, while the relative content of Cu is higher. The result indicates that part of Zr has dissolved from the diffusion–dissolution layer.

**Figure 10.**
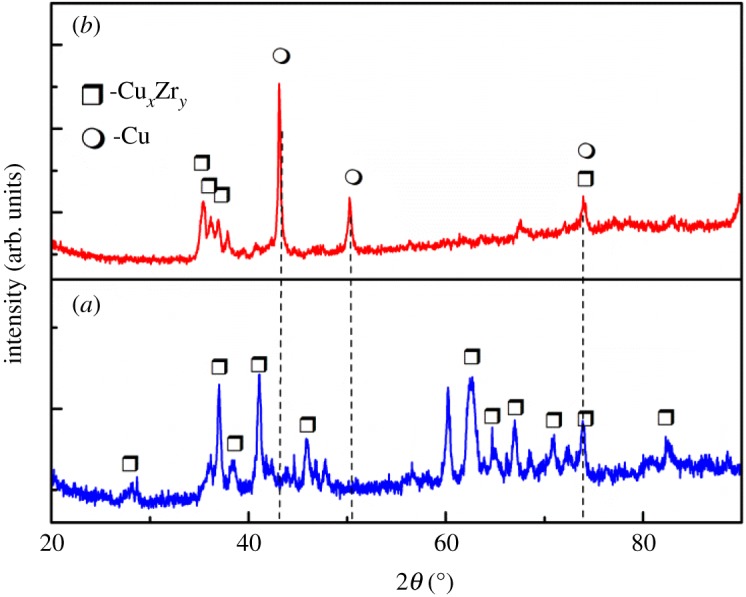
XRD patterns of (*a*) Cu−Zr alloy, and (*b*) the diffusion–dissolution layer after electrolysis at −0.80 V for 40 min.

XRD patterns of Cu−Zr alloy matrix and the diffusion–dissolution layer after electrolysis at −0.80 V for 40 min are shown in figure 10. The phase of the matrix mainly consists of Cu*_x_*Zr*_y_* intermetallic compounds, most existing in the form of Cu_10_Zr_7_. Most of the phase on the surface of the diffusion–dissolution layer was Cu with some Cu*_x_*Zr*_y_* intermetallic compounds. The results indicate that there was a small amount of Zr remaining in the diffusion–dissolution layer, which was consistent with the linear-EDS analysis in figure 9*d*.

**Figure 11.**
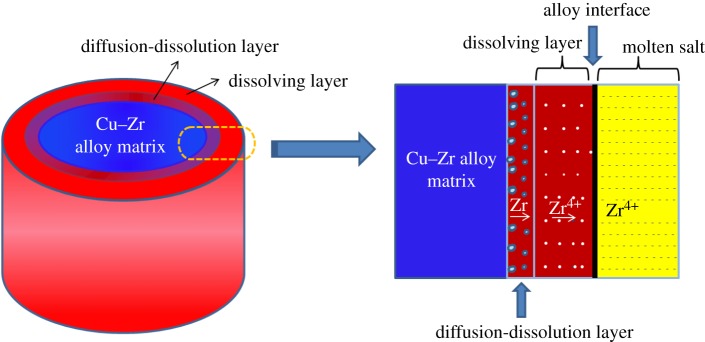
Schematic diagram of anodic dissolution model of Zr in Cu−Zr alloy.

According to the electrolysis results, the model about anodic dissolution process of Cu–Zr alloy can be inferred as follows, as shown in figure 11. The model is provided under the assumptions that the dissolution of Zr is very uniform and spreads only along the radius direction. Firstly, all the Zr on the surface dissolves and enters into the molten salt, leaving a Cu layer called ‘dissolving layer’ on the surface of the alloy. What is more, another layer between the matrix and ‘dissolving layer’, called ‘diffusion–dissolution layer’ appears. Zr diffuses from the matrix and is oxidized to Zr^4+^ on the surface of the diffusion–dissolution layer, then Zr^4+^ diffuses through the ‘dissolving layer’ and enters into the molten salt.

To sum up, anodic dissolution of Zr in the alloy can be divided into three steps. Firstly, the Zr atom in the alloy diffuses from internal to the surface of the diffusion–dissolution layer. Secondly, the Zr atom loses electrons and is oxidized, transforming into Zr^4+^ ions. Finally, Zr^4+^ ions diffuse from dissolving layer to the molten salt.

#### 3.5.2. Factors affecting the dissolution of Cu–Zr alloy

**Figure 12.**
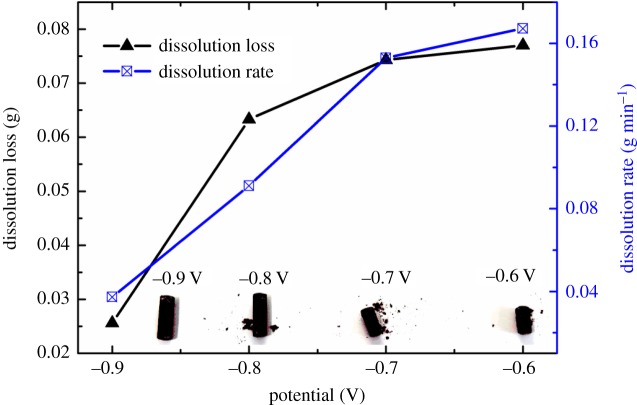
Dissolution masses and rates of Zr in Cu−Zr alloy and digital photos of the alloys after electrolysis at different potentials for 20 min.

For further understanding of the anodic dissolution of the Cu–Zr alloy, factors affecting the dissolution, such as potential and time, were investigated. The photos of the alloys after electrolysing at −0.90, −0.80, −0.70 and −0.60 V for 20 min are shown in figure 12. Excepting for the dissolved effect is not obvious at −0.90 V, there are some Cu dissolving layers falling off the other three groups.

To further determine the dissolution effects, dissolution loss and rate of Zr were calculated, as shown in figure 12. The dissolution loss is the mass loss of anode during electrolysis, that is the loss of the anode alloy before and after dissolution [17]. The dissolution rate is calculated according to the following equation:

3.1 $$v=\frac{\Delta w}{S\cdot T}$$

where *Δw* is the dissolution loss during electrolysis, g; *S* is the contact area with molten salt, cm^2^; *T* is the dissolution time, min.

From figure 12, we can see that the dissolution loss and rate increase gradually with the potential shifting positively, which can be interpreted by the polarization curve of the anode dissolution process. The more positive the potential, the higher the polarization current, the faster the dissolution rate and the more the dissolution loss. The constant potential dissolution process is not affected by Cu–Zr alloy anode passivation, which may be caused by the slow rate of constant potential dissolution.

**Figure 13.**
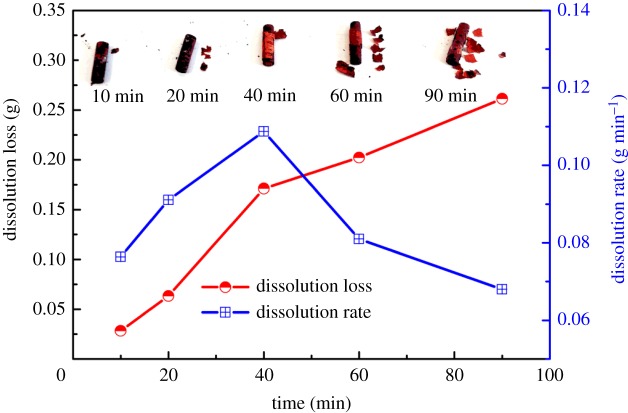
Dissolution masses and rates of Zr in Cu−Zr alloy and digital photos of the alloys after electrolysis at −0.80 V for different times.

In addition, the dissolution effect of Cu–Zr alloy after different dissolution time was studied. Figure 13 shows the dissolution rate and loss, and the digital photos of Cu–Zr alloy dissolved at −0.80 V for 10, 20, 40, 60 and 90 min. From the digital photos, we can see that the exfoliation of the rich Cu layer on the alloy surface gradually increases with the extension of dissolution time, indicating that the dissolution time will increase the dissolution of Zr. From figure 13, it also can be seen that the dissolution loss increases gradually, while the dissolution rate first increases and then decreases. This phenomenon may be because the activity of Zr on the alloy surface was large in the initial stage. With the increase in dissolution time, a rich Cu transition layer gradually appears on the surface. From figure 8, we can know that the thickness of the Cu layer has reached 100 µm when the time prolongs to 40 min.

For the spread of Zr atoms to the surface before oxidation, Cu layer and diffusion–dissolution layer have a blocking effect for the diffusion of Zr atoms. Therefore, the decline of dissolving rate at 40 min should be caused by the diffusion of Zr atom in the dissolving process.

## 4. Conclusion

In this work, the anodic dissolution potentials of Cu, Zr and Cu–Zr alloy were analysed in molten LiCl–KCl at 500°C by anode polarization curve and potentiostatic polarization curve, and the initial anodic dissolution potentials of Cu and Zr in Cu–Zr alloy were −0.52 and −0.96 V, while fast-dissolving potentials were −0.41 and −0.92 V, respectively. There was an apparent potential difference between Cu and Zr, which satisfies the potential selection principle that Zr in the alloy will be dissolved first, while Cu will be left in the anode and does not oxidize. From the potentiostatic polarization curve of Cu–Zr alloy in the potential range of −0.90 to −0.60 V, the dissolution of Zr is very significant, and the passivation of Zr and the anodic dissolution of Cu does not occur. Then potentiostatic electrolysis experiment was carried out at −0.80 V; the result shows that the Zr on the surface dissolves and enters into the molten salt, leaving a Cu layer called ‘dissolving layer’ on the surface of the alloy. What is more, another layer between the matrix and ‘dissolving layer’, called ‘diffusion–dissolution layer’, appears. That means the dissolution of Zr from Cu–Zr alloy could be well conducted. If the Cu–Zr alloy is in a liquid state, then it will be much easier. Moreover, the factors affecting the dissolution loss and rate of Zr, such as potential and time, were investigated. The dissolution loss increases with the increasing dissolution potential and time, while the dissolution rate increases with the increasing dissolution potential and decreases with the prolonging dissolution time.

## Data accessibility

This article does not contain any additional data.
